# Neutrophils and Platelets: Immune Soldiers Fighting Together in Stroke Pathophysiology

**DOI:** 10.3390/biomedicines9121945

**Published:** 2021-12-19

**Authors:** Junaid Ansari, Felicity N. E. Gavins

**Affiliations:** 1 Department of Neurology, Louisiana State University Health Shreveport, Shreveport, LA 71130, USA; 2 The Centre for Inflammation Research and Translational Medicine (CIRTM), Department of Life Sciences, Brunel University London, Uxbridge, Middlesex UB8 3PH, UK

**Keywords:** neutrophils, platelets, stroke, annexin A1, resolution, thromboinflammation

## Abstract

Neutrophils and platelets exhibit a diverse repertoire of functions in thromboinflammatory conditions such as stroke. Most cerebral ischemic events result from longstanding chronic inflammation secondary to underlying pathogenic conditions, e.g., hypertension, diabetes mellitus, obstructive sleep apnea, coronary artery disease, atrial fibrillation, morbid obesity, dyslipidemia, and sickle cell disease. Neutrophils can enable, as well as resolve, cerebrovascular inflammation via many effector functions including neutrophil extracellular traps, serine proteases and reactive oxygen species, and pro-resolving endogenous molecules such as Annexin A1. Like neutrophils, platelets also engage in pro- as well as anti-inflammatory roles in regulating cerebrovascular inflammation. These anucleated cells are at the core of stroke pathogenesis and can trigger an ischemic event via adherence to the hypoxic cerebral endothelial cells culminating in aggregation and clot formation. In this article, we review and highlight the evolving role of neutrophils and platelets in ischemic stroke and discuss ongoing preclinical and clinical strategies that may produce viable therapeutics for prevention and management of stroke.

## 1. Introduction

The understanding and the therapeutic approach to stroke has remarkably transformed in the past few decades [1]. Globally, there were approximately 6.6 million deaths attributable to stroke in 2019, which increased by 12.2% since 2010 [2]. Approximately 800,000 Americans annually suffer from stroke-related morbidity, and mortality with ischemic stroke (IS) being the most common etiology followed by hemorrhagic stroke type [2]. The recent advent of hyper-acute endovascular therapy (EVT) for large vessel occlusion in IS in the form of mechanical thrombectomy has further enhanced the neurological care and recovery [3]. The hyperacute time for the critical management of IS relies on prompt recognition of the diagnosis and urgent reperfusion/recanalization strategies. Secondary prevention strategies focus on cardiovascular and metabolic risk management such as blood pressure, glucose, cholesterol, and antithrombotic therapies.

Neutrophils and platelets are key players in ischemic brain injury and its resolution [4,5,6,7]. Resolution is the physiological ability of the body to achieve homeostasis after infection or inflammation. However, in chronic inflammation, where there is an excessive and persistent inflammatory response, the process of resolution is hampered [8,9]. Acute cerebral ischemia induces a strong immune response resulting in recruitment of several subsets of leukocytes (mainly neutrophils), activation of platelets, and coagulation cascade and upregulation of cell adhesion molecules and cytokines [10]. Neutrophils and platelets are known for their ability to produce proinflammatory/prothrombotic mediators, thereby forming an important link between inflammation and thrombosis, a phenomenon referred to as “thromboinflammation” [4,11,12]. The concept of thromboinflammation in stroke pathophysiology has gained considerable attention and traction in the last decade [4,12]. Furthermore, understanding the complex and important roles that both neutrophils and platelets play in the pathophysiology of IS continues to be a main research focus for drug discovery programs focused on finding potential therapeutics to protect against IS and for the management post-IS [10,13].

**Figure 1:**
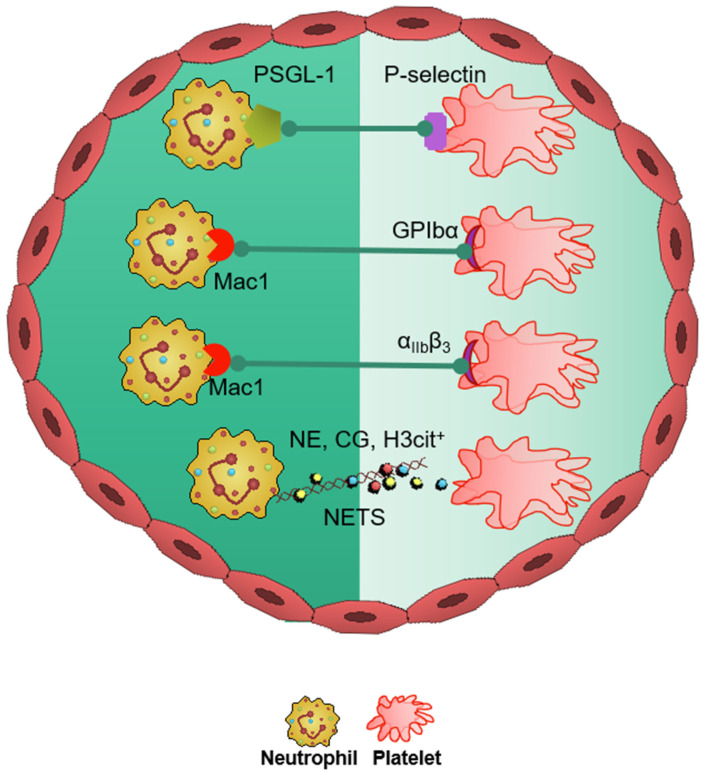
Neutrophil–platelet interactions in cerebral thromboinflammation. This figure shows the major neutrophil–platelet interactions (P-selectin glycoprotein ligand-1 [PSGL]-1-P-selectin, Mac-1-GPIbα, and Mac-1-α_IIb_β_3_) in cerebral thromboinflammation. Neutrophils also interact with platelets via productions of NETs, which are laden with various pro-thrombotic mediators such as neutrophil elastase (NE), cathepsin G (CG) and H3cit^+^ (citrullinated histone H3).

**Figure 2:**
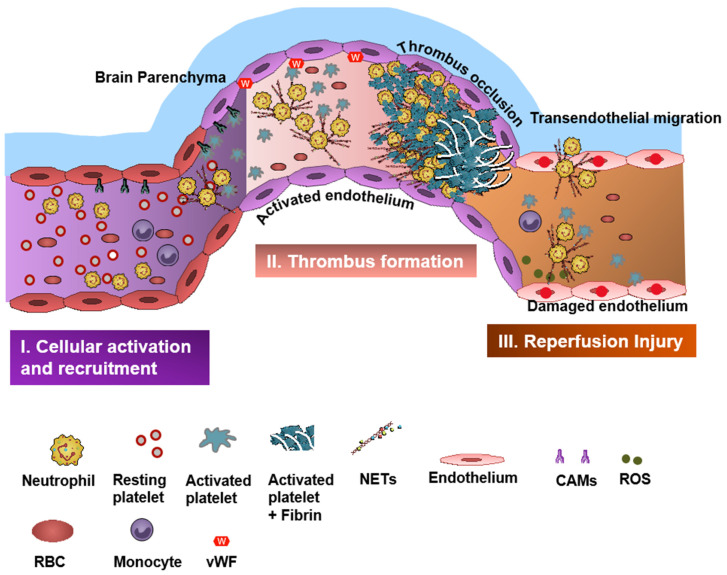
Role of neutrophil–platelet interactions in pathogenesis of stroke. (I) **Cellular activation and recruitment**. Under thromboinflammatory stress with underlying chronic inflammation there is increased recruitment and activation of cellular milieu including neutrophils and platelets into the cerebral blood vessels. This is further assisted by activation and release of cell adhesion molecules (CAMs), such as intracellular adhesion molecule and vascular adhesion molecule, and P and E selectin resulting in neutrophil activation, adherence, and rolling along the activated endothelium. Neutrophils on activation start producing various pro-thrombotic mediators such as neutrophil extracellular traps, cathepsin G, and neutrophil elastase. (II) **Thrombus formation**. The above activation and recruitment results in continuous accumulation of stimulated neutrophils, platelets, and red blood cells, and activation of the coagulation cascade. Reactive oxygen species can also enhance the coagulation cascade by inhibiting the tissue factor pathway inhibitor (TFPI). Neutrophil elastase degradation of TFPI by colocalization on NET surface. (III) **Reperfusion injury.** Reperfusion results in excessive production of pro-inflammatory and thrombotic mediators into the vessel distal to the occlusion site, resulting in microvascular dysfunction. Mainly, neutrophils produce reactive oxygen species, which further damage the endothelium and enhance neutrophil transendothelial migration. Additional tissue injury is inflicted by continuous platelet and complement system activation.

Neutrophil–platelet aggregate (NPA) formation is a well-known phenomenon and is the center of the pathogenesis of cerebral thrombus formation (Figure 1) [14]. Neutrophil-derived P-selectin glycoprotein ligand-1 (PSGL-1) and platelet P-selectin drive NPA development resulting in the activation Mac-1and LFA-1 (Mac-1 and LFA-1 are two β2 integrins expressed on neutrophils and mediate the recruitment cascade by binding to intercellular adhesive molecule 1 (ICAM-1)) [15,16]. In vivo, NPAs are also facilitated by margination of platelets and neutrophils to the periphery of blood vessels as a consequence of displacement of erythrocytes to the central part of the vessels [17]. Ischemia-reperfusion injury (I/RI), which is one of the main underlying causes of IS pathogenesis [5,18,19], further enables NPA formation and amplifies thromboinflammatory responses in IS [7,20] (Figure 2).

## 2. Neutrophils in Stroke

Neutrophils are key players in thromboinflammatory disorders including cardio- and cerebrovascular diseases [21]. These multi-lobed immune cells are amongst the first responders to migrate to the ischemic brain tissue with the zenith of invasion achieved between 48 to 72 h after ictus [13]. Here, they interact with surrounding cellular milieu including platelets, endothelial cells, microglial cells, and other brain resident cells, producing numerous pro-thrombotic mediators at the local inflammatory tissue.

The central nervous system is an immune-privileged sanctuary in which inflammatory milieu is tightly regulated to protect the neural cells from any immune response, injury, and/or death [22,23]. Neutrophils are usually restricted from trafficking into the brain parenchyma and cerebrospinal fluid (CSF) by the presence of the blood–brain barrier (BBB) [24]. Neuroinflammation seen in acute IS results in damage of BBB, making it easier for immune cells to transmigrate into the brain, with cytokines such as interleukin-1 playing significant roles in the recruitment and transmigration of neutrophils across the damaged BBB [25].

Early neutrophilia and an increased neutrophil to lymphocyte ratio in patients with IS are associated with larger infarct volumes [26] and worse functional outcomes [27,28]. Neutrophil infiltration to the infarct site is known to further dampen the sterile cerebral environment by increasing the BBB disruption [29]. Additionally, matrix-metalloproteinase (MMP)-9-positive neutrophils in IS are associated with basal lamina type IV collagen degradation and blood extravasation during hemorrhagic transformation [30].

## 3. Neutrophil Serine Proteases and Thromboinflammation

Neutrophil granule serine proteases (NSPs) have been extensively studied in inflammatory pathologies. Amongst NSPs, cathepsin G (CatG) and neutrophil elastase (NE) are particularly known to have thromboinflammatory phenotypes in various inflammatory pathologies [31,32,33,34,35]. NSPs can initiate and promote thromboinflammation in stroke by interacting with platelets and coagulation factors [11] and binding with formyl peptide receptors (FPRs) on neutrophils and platelets [32,36].

## 4. Neutrophil Extracellular Traps (NETs) and Stroke

NETosis describes a physiological response of neutrophils, when activated, to produce and extrude complexes of decondensed DNA, termed NETs [37,38]. These NETs are known to not only play a protective role in the immune response against invading pathogens, but they have also been shown to possess pro-inflammatory properties that can promote coagulation and thrombosis leading to and further exacerbating IS [5,39]. NETs are laden with prothrombotic mediators such as H3cit^+^ (citrullinated histone H3), CatG, NE and myeloperoxidase (MPO) [4,34]. Under chronic inflammatory milieu, NETosis can be detrimental and promote acute thromboinflammatory events such as IS [40,41,42,43]. Experimental studies in animal models have shown NETs can promote thromboinflammation via different NET components including H3cit^+^ and NSPs [44]. H3cit^+^ neutrophils, a pathophysiological hallmark of NETs, have been observed in all ischemic thrombi and more abundant in thrombi of cardioembolic origin compared to other etiologies [41]. Notably, a recent study revealed NETs were significantly higher in the carotid lesion site and were decorated with phosphatidylserine in thrombi [45].

Peptidylarginine deiminase 4 (PAD4) is an enzyme essential for NET formation and is known to be upregulated in thromboinflammatory disorders including IS [46]. In a model of accelerated thromboinflammation such as sickle cell disease (SCD), we found neutrophils from SCD patients increased H3Cit^+^ NETs compared to controls [4]. Furthermore, targeting SCD neutrophils with a pro-resolution molecule Annexin A1(AnxA1)_Ac2-26_ resulted in decreased H3Cit^+^ NETs from SCD neutrophils and reduced cerebral thrombosis in sickle transgenic mice [4].

## 5. Neutrophil-Dependent Oxidative Stress and IS

Neutrophils are rich sources of reactive oxygen species (ROS) and can contribute to harmful oxidative stress, which can further accelerate thromboinflammation. ROS production in the peri-infarct area has a major role in the pathogenesis of ischemic- and reperfusion-related brain injury [47,48]. ROS regulates neutrophil recruitment during inflammation by mainly inducing expression of adhesion molecules, such as vascular cell adhesion molecule-1 (VCAM-1), and can facilitate the opening of intercellular passageways to help neutrophils transmigrate to the inflammatory tissue [49]. There are multiple studies that have shown that targeting ROS production may attenuate oxidative stress and inflammation, reduce edema, and help to maintain the function and integrity of the BBB [50]. Remote ischemic conditioning and hypothermia can also attenuate oxidant stress-induced inflammation, and non-pharmacologic adjunctive ROS-targeting therapies are currently being tested to augment neurovascular protection in IS [51,52]. ROS can also enhance thromboinflammation by inhibiting the tissue factor pathway inhibitor (TFPI), which is the only physiologic inhibitor of TF activity [53].

## 6. Platelets in Stroke

Platelets are small anucleated multifaceted cells that are released from megakaryocytes [54,55], and from the bone marrow and lungs [56,57]. Their primary function is regulating hemostasis and thrombosis [58], although more recently they have been shown to play important roles in inflammation [59]. However, there is a delicate balance between the physiological and pathophysiological role of platelets due to their mediation of complex vascular responses in innate and adaptive immunity [60]. Therefore, over the years the pathophysiologic role of platelets has been studied extensively in thrombotic disorders such as myocardial infarction (MI), IS, and venous thromboembolism. This has led to the advancement of antiplatelets and anticoagulant therapies in thromboinflammatory conditions such as coronary artery disease, atrial fibrillation, and stroke [61]. Platelet production from the bone marrow is regulated by physiological homeostasis but can be adversely affected in pathophysiological conditions [59]. Thrombopoietin, secreted by the liver, is the primary growth factor and chief regulator of megakaryocytes for the platelet production, signaling via its receptor, MPL [62].

In the neurovasculature, there are distinct mechanisms of platelet-mediated thromboinflammation, which involves interaction with the neutrophils, endothelial cells, plasmatic coagulation factors, and the complement system [63,64]. In stroke, platelets and neutrophils are the first immunomodulatory cells recruited to the affected cerebral vessel where they initiate aggregation and thrombus formation [63]. The interaction of the platelets with the surrounding milieu, including circulating neutrophils, plays a significant role in regulating thromboinflammation [7,12,64]. Platelets express P-selectin on activation, which interacts with PSGL-1 to enhance neutrophil activation and recruitment at the inflammatory site. The CD40 ligand (CD40L) is found on platelets and is released on activation in the soluble circulating form, thus inducing endothelial cells to secrete chemokines and express adhesion molecules, thereby initiating a vascular inflammatory response. CD40L is also a key regulator of NPA formation and can accelerate early stages of atherosclerosis and plaque development, promote progression toward advanced atherosclerosis; and influence regulatory T cell recruitment in atherosclerosis, which is one of the main underlying causes of stroke pathogenesis [65]. Platelet PF4-dependent HIT can result in NPA formation and the development of thrombi enabling the pathogenesis of stroke [43].

Damage-associated molecular pattern molecule high-mobility group box 1 (HMGB1) is upregulated by activated platelets in multiple inflammatory diseases and has also been shown to be a critical mediator of thrombosis by regulating platelet activation, granular secretion, adhesion, and spreading [66]. HMGB1 effects on platelets seems to be mediated via platelet toll-like receptor 4 (TLR4) followed by MyD88/GC complex formation and activation of the cGMP-dependent protein kinase I (cGKI) [66]. Interestingly, platelet TLR4 also activates NET production, which can further enable stroke pathogenesis [67].

Platelet activation and aggregation resulting in thrombosis is further influenced by the high shear forces generated from the blood flow around the thrombus microenvironment [68]. The von Willebrand factor (vWF) is a key participant in the platelet-dependent thromboinflammation and stroke development [69]. Shear stress activates and brings conformational change to vWF, which then associates with platelet GPIbα (a subunit of GPIb-IX-V complex). This vWF–GPIbα interaction is crucial for initial platelet adhesion, which in turn facilitates platelet aggregation and adhesion in thrombotic events [70,71]. vWF–GPIbα interaction leads to platelet activation and results in soluble platelet agonists, such as adenosine 5′-diphosphate, adenosine 5′-triphosphate, and thromboxane A_2_ (TXA_2_), being released at the inflammatory site and shifting GPIIb/IIIa to a high-affinity state and further enabling both thrombus formation [69] and increasing the risk of IS and secondary thrombotic events post -IS [6]. Interestingly, a recent study showed that PAD4 in circulation enhances thrombosis by promoting formation of vWF-platelet string formation and reducing ADAMST13 activity [72].

## 7. Neutrophil- and Platelet-Dependent AnxA1-FPR2/ALX Resolution Axis in Stroke

Inflammation plays a key role in the pathophysiology of IS. Resolution is the ideal outcome of inflammation [4,73,74], and is defined as the mechanism to clear inflammatory influx to restore functional homeostasis. Resolution involves a tightly regulated series of events that are mediated by specialized pro-resolving mediators (SPMs) (e.g., resolvins, lipoxins, maresins, and protectins) and resolver proteins (e.g., Annexin 1 and Annexin 1-derived peptides) (10, 60–65), which are actively involved in the recovery phase of inflammation in acute and chronic conditions (8, 61, 66–68). AnxA1 and its biomimetic peptide AnxA1_Ac2-26_ have a more unique role in the resolution axis as they can target both endogenous inflammatory and pro-resolving pathways [75]. It is known that resolution is dampened in chronic inflammatory states, as shown for example by decreased levels of AnxA1 in plasma samples obtained from patients with SCD or IS compared to their respective controls [4,6]. The resolution process has also been shown to be altered or dysregulated in other inflammatory conditions, including MI, chronic kidney disease, and arthritis [6,76,77,78,79,80]. A new phase that follows resolution is known as ‘post-resolution’ in which the affected tissue develops adaptive immunity. In chronic inflammation, the post-resolution phase is not achieved due to stagnant or ‘frustrated resolution’ resulting in a delay in adaptive immunity [81]. The current research and development of novel pharmacological strategies may help in rescuing resolution biology in chronic inflammatory conditions, which in turn may help to prevent acute cerebrovascular events such as IS.

## 8. Therapeutics in Thromboinflammation

**Figure 3:**
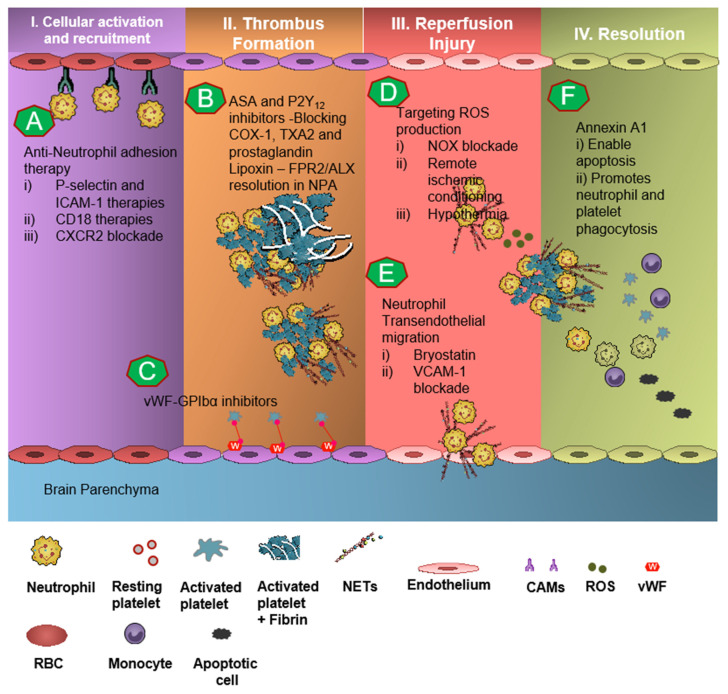
Targeting neutrophil- and platelet-dependent thromboinflammation in stroke. Schematic depiction of potential therapeutic targets to mitigate thromboinflammation in stroke. (**A**) Anti-neutrophil adhesion agents include P-selectin and intracellular adhesion molecule-1 (ICAM-1) therapies, CD18, and CXCR2 blockade. (**B**) Aspirin (ASA) and P2Y_12_ inhibition (clopidogrel and ticagrelor) inhibit the platelet activation and aggregation by antagonizing the platelet P2Y_12_ receptor. Lipoxin, an endogenous pro-resolving molecule, engages via Formyl peptide receptor-2/lipoxin-A_4_ (Fpr2/ALX) pathway and modifies neutrophil–platelet aggregate response resulting in anti-inflammatory and pro-resolving response in stroke. (**C**) Targeting von-Willebrand factor (vWF)–GPIbα interaction attenuates vWF-mediated platelet adhesion. (**D**) Targeting reactive oxygen species production by nicotinamide adenine dinucleotide phosphate (NADPH) oxidase (NOX) blockade or remote ischemic conditioning and hypothermia may attenuate oxidative stress and inflammation, reduce edema, and help to maintain the function and integrity of the blood–brain barrier (BBB) and augment neurovascular protection in stroke. (**E**) Bryostatin and vascular cell adhesion molecule (VCAM-1) blockade can inhibit neutrophil transendothelial migration. (**F**) Annexin A1 (AnxA1) and related biomimetic peptides such as Annexin A1_Ac2-26_ engage via AnxA1-Fpr2-ALX by reducing neutrophil activation and the release of pro-thrombotic mediators, regulating neutrophil H3cit^+^ (Citrullinated histone H3) production, and lastly enabling of neutrophil and platelet phagocytosis.

Due to the understanding of thromboinflammatory mechanisms in the evolution of IS, there has been significant research in drug development programs targeting neutrophil- and platelet-dependent mediators: In pre-clinical studies, engagement of the AnxA1-FPR2/ALX pathway in neutrophils as well as platelets produced significant results of mitigation and rescue of the adverse thromboinflammatory phenotype in cerebral microvessels, theoretically preventing the onset of IS as well as management of secondary I/RI-related inflammation (Figure 3) [4,6,7,75,82].

## 9. Targeting Neutrophil-Dependent Thromboinflammation

Neutrophils are the main agents of chaos in cerebral thromboinflammation and promote thrombosis and atherosclerosis via the release of various thromboinflammatory mediators as discussed above. Therefore, targeting thromboinflammatory mediators may have a critical role in management of IS by suppressing the inflammatory process and boosting neuroprotection.

Neutrophil recruitment to the ischemic site and adhesion to brain endothelial cells is enabled by P-selectin and ICAM-1 [83,84,85]. The anti-neutrophil adhesion strategy targeting P-selectin and ICAM-1 was proven to diminish neutrophil recruitment and transmigration at the site of cerebral I/R, thereby resulting in attenuation of thromboinflammation [84,85]. CD18 (leukocyte counter-ligand to endothelial intracellular adhesion molecule-1) knockout mice conferred cerebrovascular protection in a murine model of IS, but not to CD18-deficient animals with permanent middle cerebral artery occlusion, suggesting anti-neutrophil adhesion strategies should be further tested for the management of stroke [84]. However, Enlimomab, a murine ICAM-1 antibody that is known to reduce leukocyte adhesion and infarct size in experimental stroke studies, was not effective in earlier clinical trials, with more adverse events such as infections and fever compared to the placebo [86]. Studies targeting anti-E-selectin, anti-L-selectin, and chemokine receptors had no response to minimal response in animal models of experimental IS [13].

Neutrophil recruitment to the site of inflammation and stroke can result in excessive production of NSPs and reactive oxygen species (ROS), damaging the vascular as well as parenchymal structures by acting at various steps of the inflammatory cascade. Directly targeting the production of NSPs or using intracellular protease inhibitor was shown to attenuate NSP-dependent thromboinflammation [31,35]. Whereas targeting ROS production can attenuate initial as well as later stages of oxidative stress development in stroke by mitigating I/RI, restoring the BBB, and preventing neuronal death [50]. In our own work, we have shown that targeting neutrophil-dependent nicotinamide adenine dinucleotide phosphate (NADPH) oxidase may attenuate cerebrovascular thromboinflammation by inhibiting the production of H3cit^+^ neutrophils [87].

IS induces BBB permeability, thereby increasing the movement of inflammatory cells, such as neutrophils, into the brain. Enhancing and protecting the BBB against IS damage is a target of IS treatment. Bryostatin, a macrolide lactone, has been described to activate PKCδ in endothelial cells, enhance barrier integrity, block cytokine-induced barrier alterations, and potentially block neutrophil transendothelial migration [88,89]. Bryostatin treatment in an experimental model of IS resulted in improved neurological function, reduced lesion volume, and salvaged tissue compared to controls by reducing necrosis and peri-infarct astrogliosis [90].

Finally, knowing the role of NETs and PAD4 in the pathogenesis of stroke, targeting pathological NET production may be a viable approach to reduce thrombosis and stroke damage [4,87]. Our own findings have demonstrated that targeting H3cit^+^ NETs and PAD4 significantly inhibited cerebral thrombosis in vivo [4,87]. However, at present, few clinical trials have tested NET or PAD4 inhibitors in stroke management.

## 10. Targeting Platelet-Dependent Thromboinflammation

Acetylsalicylic acid (ASA), commonly known as aspirin, is one of the most common medications prescribed for primary, as well as secondary prevention of cardiovascular disease and in stroke thromboprophylaxis [91]. ASA produces clinical effect by irreversibly acetylating the active site of cyclooxygenase-1 (COX-1), thereby blocking prostaglandin and TXA_2_ synthesis, which are required for thrombus formation [92]. In a preclinical study, ASA significantly reduced cerebral leukocyte recruitment and increased endogenous levels of aspirin-triggered lipoxin, thereby inducing thromboinflammation resolution via FPR2/ALX pathway [7]. Multiple clinical trials have reported long-term secondary prevention of stroke in patients with transient of attack or IS, including non-randomized observation studies reporting a benefit of up to 80% risk reduction in recurrent stroke [91,93].

P2Y_12_ receptor is the main receptor responsible for ADP-stimulated activation of the glycoprotein IIb/IIIa receptor. Thienopyridines such as clopidogrel and ticagrelor inhibit the platelet activation and aggregation by antagonizing the platelet P2Y_12_ receptor [94]. Multiple clinical trials have shown the benefit of dual- as well as monotherapy with P2Y_12_ inhibition in stroke [95]. CHANCE and POINT revealed that the combination of clopidogrel and aspirin reduced risk of stroke in the first 90 days in patients with minor ischemic stroke or high-risk TIA, compared to those who received aspirin alone [96,97]. In a similar fashion, THALES and SOCRATES showed the benefit of ticagrelor with and without aspirin [98,99]. The recently published CHANCE-2 trial found that, in patients with minor ischemic stroke or TIA who are carriers of CYP2C19 loss-of-function alleles, the risk of stroke at 90 days was modestly lower in patients who received ticagrelor compared to clopidogrel [95,100].

Dipyridamole inhibits adenosine deaminase and platelet cAMP phosphodiesterase resulting in prevention of platelet aggregation. Multiple clinical trials have studied combination dipyridamole and aspirin for stroke management, especially the ESPS-2 trial, which showed the benefit of 25 mg of ASA twice daily and dipyridamole as equally effective for the secondary prevention of stroke and TIA [101].

Many case studies have investigated and revealed an association between high vWF levels [102] and low levels of ADAMSTS13 [103] in patients with IS. Therefore, several clinical studies have utilized strategies to inhibit vWF or enhance ADAMSTS13 in the management of stroke, including in knockout transgenic animals [69]. Most of the inhibitors targeting vWF-mediated platelet adhesion target vWF–GPIbα interaction, and are still in pre-clinical stages [104]. vWF inhibitors include monoclonal antibodies targeting vWF (e.g., 82D6A3, AJvW2, and AJW200) or targeting GPIbα 6B4 (e.g., h6B4, the nanobody ALX-0081, the aptamer ARC1779, and the recombinant GPIbα fragment GPG-290) [69].

## 11. Concluding Remarks and Future Directions

Neutrophils and platelets are seen as key players in thromboinflammation and the pathogenesis of stroke. The emerging role of neutrophil-derived serine proteases, extracellular traps, and ROS in the cerebrovascular thromboinflammation has created an immense opportunity for the development of translational research. Current evidence suggests the dampening of resolution pathways/mediators in thromboinflammatory conditions such as stroke, therefore leading to an unchecked and persistent burden of pro-inflammatory milieu. The ongoing research including our own will be instrumental in developing viable drug discovery programs that target proteins and pathways involved in pathophysiological settings (such as H3Cit^+^ NETs and PAD4) to enable inflammation resolution. In a similar fashion, platelet-dependent thromboinflammation has and can be effectively targeted by inhibiting the pathophysiological activation of vWF–GPIbα interaction, P2Y_12_, CD40L, and TLR4. Additional targeting and modulating NPA formation can mitigate the secondary complications of chronic thromboinflammation such as stroke. Finally, exploiting endogenous protective mechanisms and pathways (e.g., the AnxA1/FPR2/ALX pathway) in neutrophils and platelets, thereby enabling the resolution of thromboinflammation, is going to be impactful in developing novel and potent therapies against stroke and will help drive effective pre-clinical and clinical therapeutic studies.

## Data Availability

Not applicable.
